# Investigation of the role of *SDHB* inactivation in sporadic phaeochromocytoma and neuroblastoma

**DOI:** 10.1038/sj.bjc.6602202

**Published:** 2004-10-26

**Authors:** D Astuti, M Morris, C Krona, F Abel, D Gentle, T Martinsson, P Kogner, H P H Neumann, R Voutilainen, C Eng, P Rustin, F Latif, E R Maher

**Affiliations:** 1 Section of Medical and Molecular Genetics, Department of Paediatrics and Child Health, University of Birmingham, The Medical School, Edgbaston, Birmingham B15 2TT, UK; 2 Cancer Research UK Renal Molecular Oncology Research Group, University of Birmingham, The Medical School, Edgbaston, Birmingham B15 2TT, UK; 3 Department of Clinical Genetics, Gothenburg University, Sahlgrenska University Hospital/Ostra, S-416 85 Gothenburg, Sweden; 4 Childhood Cancer Research Unit, Department of Woman and Child Health, Karolinska Institute, Karolinska Hospital, S-171 76 Stockholm, Sweden; 5 Medizinische Universitatsklinik, Hugstetter Str. 55, D-79106 Freiburg, Germany; 6 Department of Paediatrics, Kuopio University Hospital, FIN-70211 Kuopio, Finland; 7 Department of Pathology, Haartman-Institute, FIN-00014 University of Helsinki, Helsinki, Finland; 8 Clinical Cancer Genetics and Human Cancer Genetics Programs, Comprehensive Cancer Center, the Division of Human Genetics, Department of Internal Medicine, The Ohio State University, Columbus, OH, USA; 9 INSERM U393 Handicaps Génétique de l'Enfant, Hôpital Necker-Enfants Malades, 149, rue de Sèvres, 75015 Paris, France

**Keywords:** SDHB, methylation, neuroblastoma, phaeochromocytoma

## Abstract

Germline mutations in the succinate dehydrogenase (SDH) (mitochondrial respiratory chain complex II) subunit B gene, *SDHB*, cause susceptibility to head and neck paraganglioma and phaeochromocytoma. Previously, we did not identify somatic *SDHB* mutations in sporadic phaeochromocytoma, but *SDHB* maps to 1p36, a region of frequent loss of heterozygosity (LOH) in neuroblastoma as well. Hence, to evaluate *SDHB* as a candidate neuroblastoma tumour suppressor gene (TSG) we performed mutation analysis in 46 primary neuroblastomas by direct sequencing, but did not identify germline or somatic *SDHB* mutations. As TSGs such as *RASSF1A* are frequently inactivated by promoter region hypermethylation, we designed a methylation-sensitive PCR-based assay to detect *SDHB* promoter region methylation. In 21% of primary neuroblastomas and 32% of phaeochromocytomas (32%) methylated (and unmethylated) alleles were detected. Although promoter region methylation was also detected in two neuroblastoma cell lines, this was not associated with silencing of *SDHB* expression, and treatment with a demethylating agent (5-azacytidine) did not increase SDH activity. These findings suggest that although germline *SDHB* mutations are an important cause of phaeochromocytoma susceptibility, somatic inactivation of *SDHB* does not have a major role in sporadic neural crest tumours and *SDHB* is not the target of 1p36 allele loss in neuroblastoma and phaeochromocytoma.

Neuroblastoma and phaeochromocytoma are the most common neural crest-derived tumours in children and adults, respectively. Neuroblastoma is clinically variable with some tumours demonstrating spontaneous regression after little or no therapy, while in other cases distant metastases are present at diagnosis. Familial neuroblastoma is rare and major susceptibility genes have not yet been isolated. Phaeochromocytomas usually present with hypertension and 90% are benign. Germline mutations in the *RET, VHL, SDHB* and *SDHD* genes are important causes of phaeochromocytoma susceptibility and phaeochromocytoma may also rarely (<1%) complicate neurofibromatosis type 1 (reviewed by Maher and Eng 2002, Eng *et al*, 2003). Human cancer genetics provides many examples of how the identification of a rare inherited cancer susceptibility gene has provided insights into the pathogenesis of sporadic cases. However, exceptions exist: although von Hippel–Lindau disease is a major cause of familial clear cell renal carcinoma (cRCC) and somatic inactivation of the *VHL* tumour suppressor gene (TSG) occurs in most sporadic cRCC (Gnarra *et al*, 1994; Foster *et al*, 1994; Herman *et al*, 1994; Clifford *et al*, 1998), somatic VHL inactivation by mutation or methylation of the promoter region is infrequent (<5%) in sporadic phaeochromocytomas. In addition, although both phaeochromocytoma and medullary thyroid cancer are major features of MEN 2A and MEN 2B and somatic *RET* mutations are common in sporadic medullary thyroid cancer (Eng *et al*, 1994, 1995), somatic *RET* mutations are found in only 10% of sporadic phaeochromocytomas (Eng *et al*, 1995; Hofstra *et al*, 1996). Thus, *VHL* and *RET* appear to have only a minor role in the pathogenesis of sporadic phaeochromocytoma.

The *SDHB* and *SDHD* genes encode two (of four) subunits of the mitochondrial respiratory chain complex II (succinate dehydrogenase: SDH). Germline mutations in *SDHB* and *SDHD*, in addition to causing phaeochromocytoma, may also predispose to the development of head and neck paragangliomas (most commonly carotid body tumours) (Baysal *et al*, 2000; Gimm *et al* 2000, Astuti *et al*, 2001a, 2001b, 2003; Gimenez-Roqueplo *et al*, 2002; Neumann *et al*, 2002, Benn *et al*, 2003; Leube *et al*, 2004). Familial phaeochromocytoma or head and neck paraganglioma (HNPGL) kindreds with germline *SDHD* mutations demonstrate parent-of-origin effects on penetrance (Baysal *et al*, 2000; Astuti *et al*, 2001a). In contrast, *SDHB* mutations show no evidence of genomic imprinting effect. *SDHB* maps to 1p36, a region of frequent allele loss in many tumour types including neuroblastoma and phaeochromocytoma (Martinsson *et al*, 1997; Maris and Matthay, 1999; Benn *et al*, 2000; Ejeskar *et al*, 2001). Previously, we did not detect somatic *SDHB* mutations in 24 sporadic phaeochromocytomas (Astuti *et al*, 2001b) and this has been confirmed by others (Benn *et al*, 2003). However, studies of a number of TSGs have established a paradigm in which specific TSGs can be inactivated frequently by *de novo* promoter methylation but rarely by somatic mutations (Dammann *et al*, 2000; Agathanggelou *et al*, 2001; Burbee *et al*, 2001; Morrissey *et al*, 2001). In keeping with this, we have reported frequent *RASSF1A* hypermethylation in neuroblastoma and phaeochromocytoma (Astuti *et al*, 2001c). These findings prompted us to investigate whether *SDHB* promoter methylation occurred in neuroblastoma and phaeochromocytoma.

## MATERIALS AND METHODS

### Clinical material

DNA were extracted from frozen primary tumour tissue from (a) 35 sporadic phaeochromocytomas without evidence of germline or somatic *SDHB* mutations (four tumours were from patients with von Hippel – Lindau disease and three from patients with MEN2A) and one phaeochromocytoma with a germline *SDHB* mutation was analysed (Astuti *et al*, 2001b); (b) 46 neuroblastomas and (c) from corresponding normal tissue samples (fibroblast or blood) were analysed. Approval from the appropriate Institutional Review Boards and informed consent from all patients were obtained. Most of this tumour material has been described earlier (Martinsson *et al*, 1997; Ejeskär *et al*, 1998; Astuti *et al*, 2001c).

### Bisulphite modification and methylation-specific PCR (MSP)

**Figure 1:**
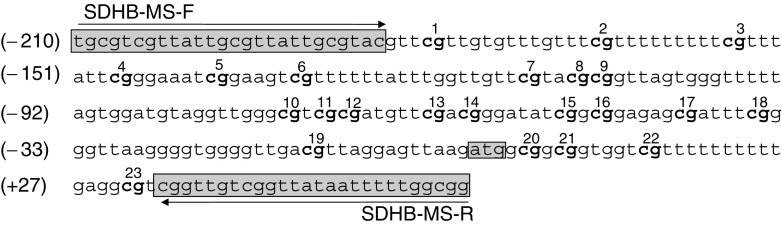
*SDHB* promoter sequence. In boxes shaded in light grey are the MSP primer sequences.CpG islands are in bold and numbered from 1 to 23. The numbers in brackets are nucleotide position in relation to the ATG start codon (highlighted in light grey).

Bisulphite DNA modification was performed as described previously (Herman *et al*, 1996). Briefly, 0.5–1.0 *μ*g of genomic DNA was denatured in 0.3 M NaOH for 15 min at 37°C and then unmethylated cytosine residues were sulphonated by incubation in 3.12 M sodium bisulphite (pH 5.0) (Sigma) 5 mM hydroquinone (Sigma) in a thermocycler (Hybaid) for 30 s at 99°C/15 min at 50°C for 20 cycles. The sulphonated DNA was recovered using the Wizard DNA clean-up system (Promega) in accordance with the manufacturer's instructions. The conversion reaction was completed by desulphonating in 0.3 M NaOH for 10 min at room temperature. The DNA was ethanol precipitated and resuspended in water. Methylation-specific PCR was performed using specific primers designed to amplify methylated and unmethylated putative SDHB promoter sequences (Au *et al*, 1995; GeneBank accession No. U17296): unmethylated –specific, 5′-TGTGTTGTTATTGTGTTATTGTGTAT-3′ (forward) and 5′-CCACCAAAAATTATAACCAACAACCA-3′ (reverse) and methylated –specific, 5′-TGCGTCGTTATTGCGTTATTGCGTAC-3′ (forward) and 5′-CCGCCAAAAATTATAACCGACAACCG-3′ (reverse) (Figure 1

). Taq DNA polymerase (Gibco) was added after a ‘hot start’ at 95°C for 5 min. Amplification was carried out for 35 cycles at an annealing temperature of 53°C for the unmethylated specific primers and 61°C for the methylated specific primers on Omn-E (Hybaid) DNA thermal cycler. The expected sizes of the PCR products for both unmethylated and methylation-specific amplifications were 269 bp.

### Cloning and sequencing of PCR products

The PCR products containing bisulphite-resistant cytosines were purified using PCR product purification kit (Qiagen) and ligated into the pGEM-T easy vector system (Promega), according to the manufacturer's instructions. Several clones were then isolated and sequenced using ABI 377 DNA analyser (Applied Biosystem).

#### Mutation analysis

*SDHB* mutation analysis was performed by direct sequencing of coding sequence amplicons as previously described (Astuti *et al*, 2001b). The GenBank accession number for SDHB exons 1–8 are: U17296, U17880, U17881, U17882, U17883, U17884, U17885 and U17886. Sequence analysis was performed on an ABI PRISM 3100 DNA Sequencer (Applied Biosystems). The sequencing products were compared to the *SDHB* reference sequence NM_003000.

### Cell culture and Western blot analysis

Two neuroblastoma cell lines (SK-N-AS and SK-N-SH) purchased from ATCC were grown in Dulbecco's modified eagle medium, supplemented with 10% foetal calf serum. Demethylation was performed by the addition of 2 *μ*M 5-aza-2-deoxycytidine to the growth medium. This latter was replenished with fresh medium after 3 days. On the fifth day of treatment, total protein was extracted in NETS lysis buffer (150 mM NaCl, 50 mM Tris (pH 8) 5 mM EDTA, 1% NP40) containing 3 mM PMSF, 20 *μ*g ml^−1^ aprotonin and 10 *μ*g ml^−1^ leupeptin. Following homogenisation and incubation on ice for 10 min, lysates were centrifuged for 15 min at 14 000 rpm/4°C and stored at −20°C.

Protein samples (20 *μ*g each) were separated on sodium dodecyl sulphate-10.5% polyacrylamide gel and electroblotted to transblot polyvinylidene difluoride membrane (Hybond-P; Amersham Bioscience, Chalfont St Giles, UK). Anti-SDHB (Molecular Probes, clone: 21A11-AE7) at 2.5 *μ*g ml^−1^ was applied followed by rabbit anti-mouse immunoglobulin-peroxidase conjugate. Visualisation was carried out by the enhanced chemiluminescence detection system (ECL-plus; Amersham Bioscience). The filter was stained with India ink for standardisation, and quantification was performed using a Bio-Rad imaging densitometer with Quantity One software.

#### Enzyme assays

Succinate cytochrome *c* reductase (complex II and III) and quinol cytochrome *c* reductase (complex III) activities were spectrophotometrically measured in neuroblastoma cell line homogenates as previously described (Rustin *et al*, 1994).

### Loss of heterozygosity (LOH) analysis

Assessment of neuroblastoma samples for 1p loss of heterozygosity (LOH) has been reported previously (Martinsson *et al*, 1995, 1997; Ejeskär *et al*, 2001). The 1p allele status of the phaeochromocytoma samples was investigated using a panel of 14 polymorphic microsatellite markers, including 1pter-D1S243, D1S1646, D1S1635, D1S434, D1S1597, D1S228, D1S552, D1S1676, D1S1622, D1S2134, D1S1661, D1S1596, D1S551 and D1S435-1cen. Primer sequences are available from the Genome Database (http://gdbwww.gdb.org). The PCR products were electrophoresed on an 8% urea – polyacrylamide gel and were visualised by silver staining. Allelic loss was considered to have occurred in tumour samples when there was a 50% or greater reduction in signal intensity of an allele in tumour DNA compared to normal DNA.

#### Statistical analysis

Comparisons were made by Fisher's exact test (two tailed). *P*-values of 0.05 were taken as statistically significant.

## RESULTS

### *SDHB* methylation and mutation status in neuroblastoma

Direct sequencing of the *SDHB* coding exons and flanking sequences in 46 neuroblastoma tumours was performed. No pathogenic mutations were detected, although a number of known sequence variants and deviations from reference sequence were detected. One silent heterozygous SNP (18A>C) was identified in a stage 4 neuroblastoma with a fatal outcome of the disease. Some variations from the reference sequence (c.-16delG, IVS3-(18-19) insA, IVS3-(24-25)insA, IVS7+4delA, and IVS8+(19-20)insT) were present in homozygous form in all samples including the control, and they are thus likely to be errors in the reference sequence. A trinucleotide repeat, TTC_n_, with the most 3′ nucleotide located 14 bases upstream of exon 5 was found to be polymorphic. The number of repeats varied between 6 and 10 with 8 repetitions being the most common allele. Of 94 neuroblastoma tumour samples tested, 91 were homozygous (or hemizygous) TTC_8_ compared to 98 out of 99 control samples.

**Figure 2:**
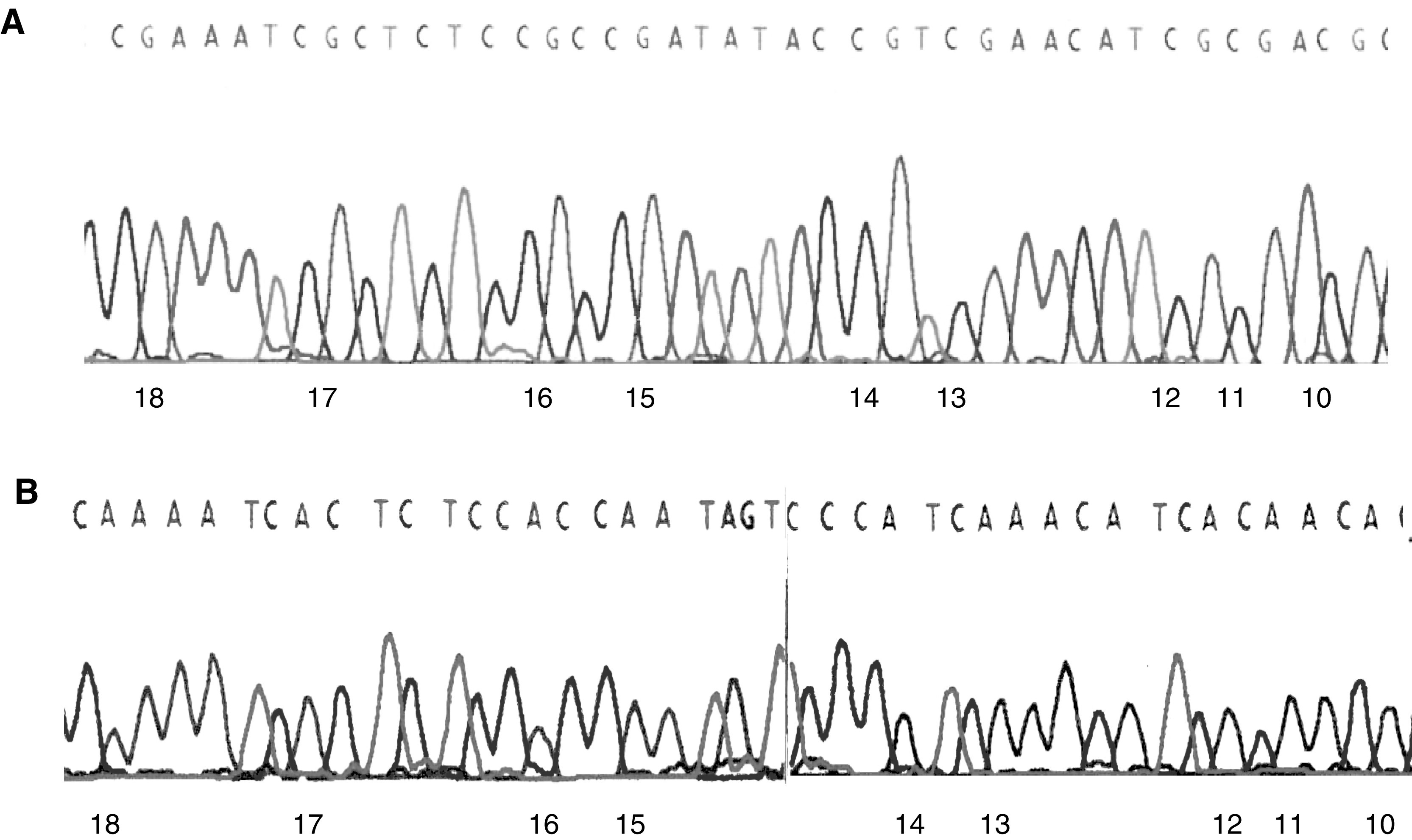
(**A**) Partial chromatogram of cloned MSP product from a methylated neuroblastoma tumour (St158T). Methylated cytosine appear as a G signal in the complementary strand. The number indicates the position of CpG sites. (**B**) Similar chromatogram obtained from an unmethylated neuroblastoma tumour (St111T).

*SDHB* promoter methylation status was investigated in 46 primary neuroblastoma tumours. In all, 22% (10 out of 46) of the neuroblastomas demonstrated *SDHB* CpG island promoter methylation by MSP analysis compared to 0 of 20 normal control blood samples. Sequencing of the MSP product (10 individual clones from two methylated tumours) demonstrated that 22 of the 23 CpG dinucleotides in the fragment were methylated in each tumour (Figure 2

). In each tumour with SDHB methylation, unmethylated alleles were also detected so there was no evidence of complete methylation. There was no significant difference between the frequency of *SDHB* promoter methylation in neuroblastoma tumours with and without 1p36 allele loss and no correlation with 3p allele loss, 17q gain or N-myc amplification status. Furthermore, there was no association between partial *SDHB* promoter methylation and tumour stage (21% of stage 1, 2 and 4S tumours, and in 27% of stage 3 and 4 tumours).

### 1pLOH analysis and *SDHB* promoter methylation in sporadic phaeochromocytomas

**Figure 3:**
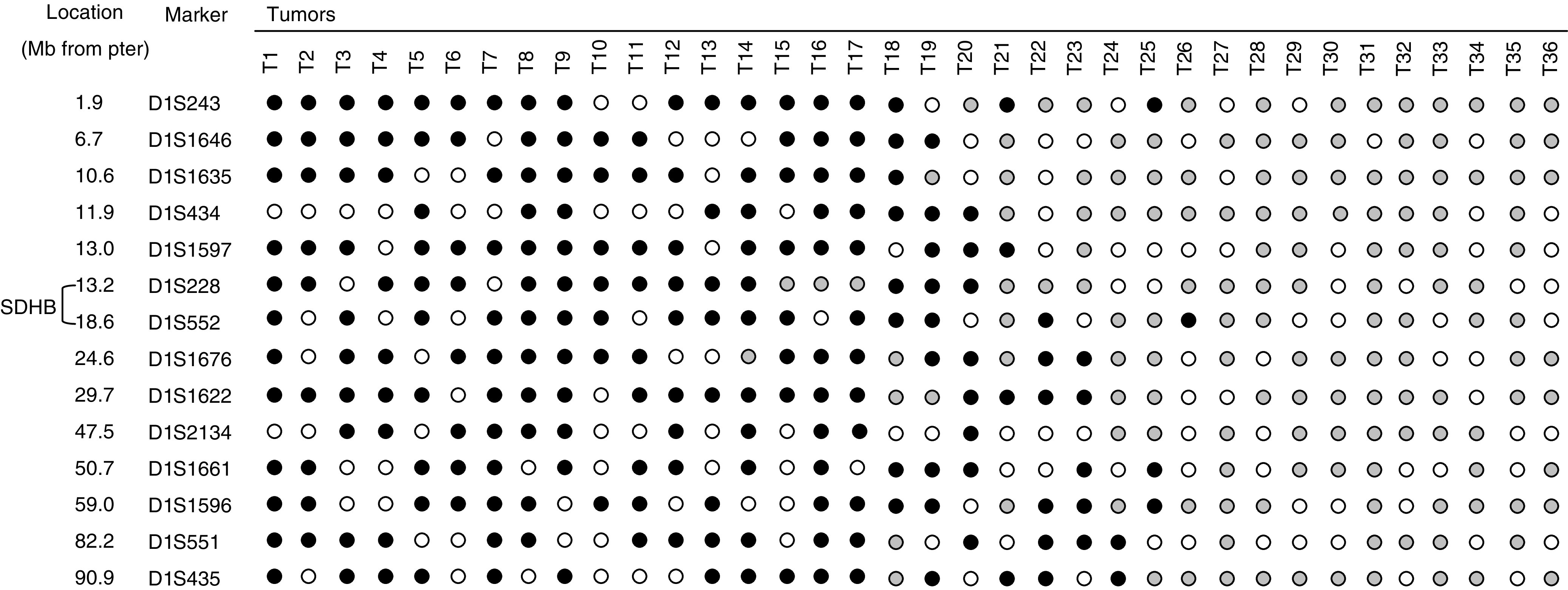
Summary of chromosome 1p loss of heterozygosity analysis in phaeochromocytomas. Filled circles indicate LOH; shaded circles indicate retention of heterozygosity and open circles indicate noninformative cases. Microsatellite markers are ordered from telomere to centromere (Genome Browser-Human assembly, July 2003; http://genome.ucsc.edu).

Previously we did not find evidence of somatic *SDHB* mutations in sporadic phaeochromocytomas (Astuti *et al*, 2001b). However, to investigate further the potential role of *SDHB* in the pathogenesis of phaeochromocytoma, we determined the frequency, extent and patterns of 1p allele loss in 36 sporadic phaeochromocytomas using 14 polymorphic microsatellite markers mapping to 1p22–1p36. In all, 75% (27/36) of tumours demonstrated LOH at one or more 1p locus (Figure 3

). A total of 10 tumours demonstrated LOH at all informative markers and nine demonstrated retention at all informative markers. *SDHB* maps between D1S228 and D1S552 (∼1.7 MB from D1S552) and LOH was observed in 54 and 64%, respectively, of informative tumours at these flanking markers. A phaeochromocytoma sample with a germline *SDHB* mutation demonstrated 1p allele loss (and no methylation, T12 – see later) consistent with a ‘two hits’ model of tumourigenesis.

**Figure 4:**
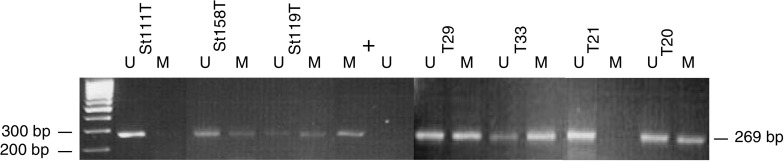
MSP analysis of *SDHB* methylation in sporadic neuroblastoma (St111T, St158T and St119T) tumours and in sporadic phaeochromocytoma (T29, T33, T21 and T20) tumours. Bisulphite-modified DNA was amplified with primer pair specific for unmethylated (U) and methylated (M) alleles as described in the text. *In vitro* methylated DNA was used as a positive control (+) for amplification with methylated DNA-specific primers.

*SDHB* promoter methylation was detected in nine out of 28 (32%) of phaeochromocytomas analysed by MSP (all matching blood DNA samples were unmethylated) (Figure 4

). In addition to methylation-specific PCR products, unmethylated-specific products were also amplified from each of the nine ‘methylated tumours’ consistent with partial methylation in tumours and/or the presence of contaminating normal tissue in the tumour samples. Sequencing of the MSP product (10 individual clones from each of two methylated phaeochromocytomas) demonstrated methylation at 21 of the 23 CpGs analysed (data not shown). There was no difference between the frequency of LOH close to *SDHB* in phaeochromocytoma with and without *SDHB* promoter methylation (75 *vs* 57% respectively, *P*=0.42).

### Functional significance of *SDHB* promoter region methylation

**Figure 5:**
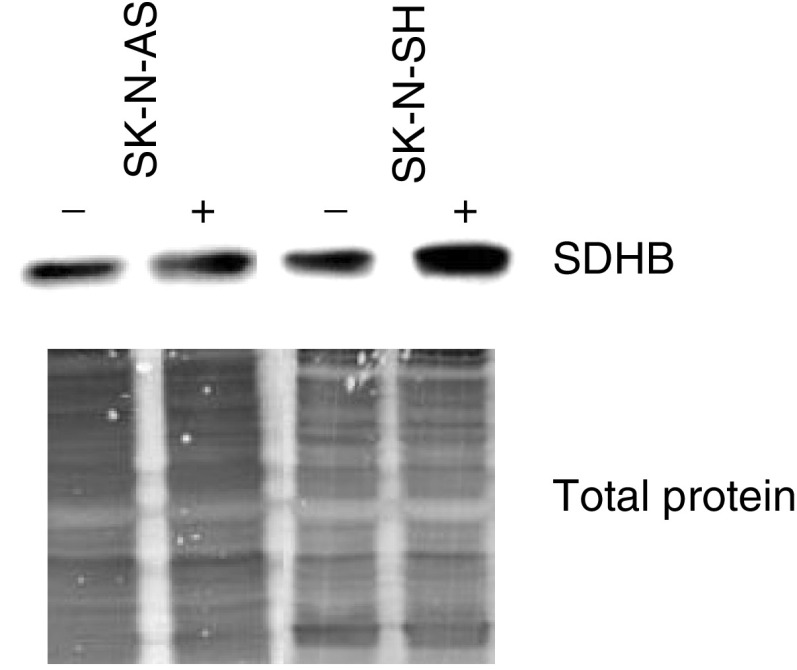
Expression analysis of *SDHB* gene in neuroblastoma cell line SK-N-AS and SK-N-SH, before (−) and after (+) 5 aza-2-deoxycytidine treatment. Western blotting was performed essentially as described in the Material and Methods. Indian ink staining was included as a loading control.

To investigate the possible functional significance of this partial promoter methylation, we screened eight neuroblastoma cell lines and identified two (SK-N-SH and SK-N-AS) with partial *SDHB* methylation by MSP. We then treated these two cell lines with the demethylating agent, 5-azacytidine, for 5 days and evaluated the effect on *SDHB* protein expression. Before treatment, SDHB was readily detectable and following treatment with 5-azacytidine, there were small increases in *SDHB* protein expression (SDHB protein (up to three- and two-fold in SK-N-SH and SK-N-AS cells respectively) (Figure 5

). However, the relatively small changes in SDHB expression were not associated with evidence of enhanced SDH enzyme activity. Thus, the ratio of quinol cytochrome *c* reductase (complex III) (QCCR) to succinate cytochrome *c* reductase (complex II and III) (SCCR) enzyme activities was not abnormally increased prior to treatment with 5-aza-2-deoxycytidine, and there was no reduction in QCCR/SCCR ratio after demethylation (SK-N-SH cell line: Pretreatment QCCR/SCCR ratio=2.13, post-treatment 3.7; SK-N-AS cell line, pretreatment QCCR/SCCR ratio=2.85, post-treatment 3.44; controls (lymphoblastoid cell lines: QCCR/SCCR ration=3.1±0.3).

## DISCUSSION

Neuroblastomas and phaeochromocytomas are the most common neural crest-derived tumours in children and adults, respectively, and it is of interest to compare the molecular pathology of the two tumours. The molecular pathology of sporadic neuroblastomas has been investigated extensively. Frequent alterations include N-myc amplification (20–25%) and gain of genetic material at 17q23 – qter (−50% of tumours). Neuroblastoma suppressor genes have been mapped by LOH studies to 1p36 (30–35% of primary tumours show LOH), 11q23 (44%) and 14q23l – qter (22%) (reviewed in Maris and Matthay, 1999). In addition to these well-defined genetic alterations, we and others have demonstrated that epigenetic TSG inactivation may be a feature of neuroblastoma. Thus, *CASP8* promoter methylation has been reported in ∼50% of neuroblastomas by us and other (Teitz *et al*, 2000; Astuti *et al*, 2001c; Harada *et al*, 2002a) and *RASSF1A* promoter methylation also occurs frequently (52–55% (Astuti *et al*, 2001c; Harada *et al*, 2002b). However, Harada *et al* (2002b) detected no or little promoter methylation of *p16*^*INK4A*^ (0%), *MGMT* (0%), *RARB* (0%), *DAPK* (0%), *APC* (0%), *CDH13* (0%), *CDH1* (6%) and *GSTP1* (3%) in primary neuroblastoma tumours. These genes have all demonstrated promoter methylation in other cancer types and so most TSGs analysed to date do not show promoter methylation in neuroblastoma.

Although there is compelling evidence for a major neuroblastoma suppressor gene on 1p, to date, a major 1p36.2 – p36.3 neuroblastoma suppressor gene has not been identified (Ejeskär *et al*, 1999; Jogi *et al*, 2000; Abel *et al*, 2002). We did not detect somatic *SDHB* gene mutations in neuroblastoma and we could not demonstrate evidence for epigenetic inactivation. In addition, we note that the critical neuroblastoma suppressor gene interval defined by Ejeskar *et al* (2001) (D1S508 to D1S244) and the 500 kb 1p36.2 – p36.3 homozygous deletion in a neuroblastoma cell line reported by Ohira *et al* (2000), both map >4 Mb telomeric to *SDHB*. *CASP8* and *RASSF1A* methylation in neuroblastoma is associated with transcriptional downregulation, but in contrast *SDHB* promoter methylation did not impair SDH enzyme activity. We note that despite tumour-specific *WT1* promoter methylation in primary breast cancer, WT1 protein is still expressed in these tumours (Loeb *et al*, 2001). While MSP provides a sensitive technique for detecting promoter methylation in tumour samples, the ability to detect low levels of methylation, in only a subset of tumour cells, can exaggerate the frequency of promoter methylation.

Even though germline *SDHB* mutations are an important cause of phaeochromocytoma susceptibility (Astuti *et al*, 2001b; Neumann *et al*, 2002), we did not identify somatic *SDHB* mutations in phaeochromocytoma so far. Similarly, germline mutations in the *VHL* TSG are an important cause of phaeochromocytoma susceptibility, but somatic *VHL* mutations are rare in phaeochromocytoma (Eng *et al*, 1995; Woodward *et al*, 1997). The finding of 1p LOH in a phaeochromocytoma with a germline *SDHB* mutation is consistent with a two hit hypothesis of tumorigenesis and the frequent occurrence of 1p LOH in sporadic phaeochromocytomas without *SDHB* mutations suggested that in some cases *SDHB* inactivation could occur by a combination of LOH and *SDHB* promoter methylation. However Benn *et al* (2000) have suggested that there were at least two distinct intervals (three possible regions) of 1p LOH in phaeochromocytoma. *SDHB* maps outside the most telomeric distinct interval (PC1, D1S243 to D1S244) but is contained within the second interval (D1S228 to >40 cM centromeric). In our LOH studies, 10 tumours with partial 1p LOH had no LOH at D1S228 but LOH at more centromeric markers. *SDHB* maps ∼4 Mb centromeric to D1S228 (http://genome.ucsc.edu/cgi-bin /hgGateway) so LOH studies did not exclude *SDHB* being implicated in phaeochromocytoma tumorigenesis. As for chromosome 3p, multiple TSGs may map to 1p. We note that in several tumours there were complicated patterns of LOH with areas of LOH flanking a marker with retention of heterozygosity. Such patterns may reflect the involvement of multiple TSGs in a single tumour. Although we detected evidence for partial *SDHB* promoter methylation using the sensitive MSP technique in a subset of phaeochromocytomas, this degree of methylation did not impair SDH activity (for comparison, Gimenez-Roqueplo *et al* (2002) found a mean QCCR/SCCR ratio of >200 in phaeochromocytomas with *SDHB* mutations and 2.7 in phaeochromocytomas without *SDHB* mutations).

The mechanism whereby germline *SDHB* mutations promote tumorigenesis is uncertain. *SDHB* inactivation may lead to upregulation of a wide range of hypoxia-inducible genes (Gimenez-Roqueplo *et al*, 2002). Activation of hypoxia-responsive pathways may have an important role in cancer development and may be caused by local tissue hypoxia or result from genetic mechanisms (An *et al*, 1998; Maxwell *et al*, 1999; Zundel *et al*, 2000). However, germline *VHL* mutations that cause phaeochromocytomas and not other features of VHL disease retain the ability to regulate hypoxia-inducible factor HIF-1 and HIF-2 (Clifford *et al*, 2001; Hoffman *et al*, 2001). Mitochondrial dysfunction may reduce apoptosis and promote tumorigenesis (Green and Reed, 1998), and is another mechanism by which *SDHB* inactivation could promote tumorigenesis. Further work is required to define the precise mechanism of *SDHB* tumour suppression and how these explain the restricted phenotype of *SDHB*-associated tumours and the lack of evidence for a role of somatic *SDHB* inactivation in the pathogenesis of sporadic phaeochromocytomas.
